# Cryptic species diversity in polypores: the *Skeletocutis nivea* species complex

**DOI:** 10.3897/mycokeys.36.27002

**Published:** 2018-07-18

**Authors:** Aku Korhonen, Jaya Seelan Sathiya Seelan, Otto Miettinen

**Affiliations:** 1 Finnish Museum of Natural History, University of Helsinki, PO Box 7, 00014 University of Helsinki, Finland; 2 Natural Resources Institute Finland (Luke), PO Box 2 (Latokartanonkaari 9), FI-00791, Helsinki, Finland; 3 Mycology and Pathology Laboratory, Institute for Tropical Biology and Conservation (ITBC), Universiti Malaysia Sabah, 88400 Kota Kinabalu, Sabah, Malaysia

**Keywords:** cryptic species, fungal taxonomy, Incrustoporiaceae, phylogenetic species, polypores

## Abstract

We propose a taxonomic revision of the two closely related white-rot polypore species, *Skeletocutis nivea* (Jungh.) Jean Keller and *S. ochroalba* Niemelä (Incrustoporiaceae, Basidiomycota), based on phylogenetic analyses of nuclear ribosomal internal transcribed spacer (ITS) and translation elongation factor EF-1α sequences. We show that prevailing morphological species concepts of *S. nivea* and *S. ochroalba* are non-monophyletic and we delineate new species boundaries based on phylogenetic inference. We recognise eleven species within the prevailing species concept of *S. nivea* (*S. calida* **sp. nov.**, *S. coprosmae* comb. nov., *S. futilis* **sp. nov.**, *S. impervia* **sp. nov.**, *S. ipuletii* **sp. nov.**, *S. lepida* **sp. nov.**, *S. nemoralis* **sp. nov.**, *S. nivea* sensu typi, *S. semipileata* comb. nov., *S. unguina* **sp. nov.** and *S. yuchengii* **sp. nov.**) and assign new sequenced epitypes for *S. nivea* and *S. semipileata.* The traditional concept of *S. ochroalba* comprises two independent lineages embedded within the *S. nivea* species complex. The Eurasian conifer-dwelling species *S. cummata* **sp. nov.** is recognised as separate from the North American *S. ochroalba* sensu stricto. Despite comprehensive microscopic examination, the majority of the recognised species are left without stable diagnostic character combinations that would enable species identification based solely on morphology and ecology.

## Introduction

Species delimitation in macrofungi has traditionally been based on morphology of fruiting bodies. Yet, their structure is often relatively simple and taxonomically useful characters are scarce. Even with rigorous microscopic inspection of hyphal structures and spores, true species diversity in macrofungi appears to be concealed by limited morphological resolution.

In polypores (a form group of primarily wood-decaying Basidiomycota with poroid hymenophores), numerous DNA-based studies have reported unaccounted species diversity within previously recognised morphospecies: e.g. *Antrodia crassa* (Spirin et al. 2015), *Heterobasidion insulare* (Chen et al. 2014, Dai and Korhonen 2009), *Laetiporus sulphureus* (Banik et al. 2010, Vasaitis et al. 2009), *Phellinus igniarius* (Tomovský et al. 2010, Zhou et al. 2016) and *Porodaedalea pini* (Brazee and Lindner 2013). In the above-mentioned cases, the newly discovered species can be, and mostly were, characterised morphologically. It should be noted however that much of morphological taxonomy, including our work, is conducted today as follows: first sequence your material, postulate phylogenetically supported species and then see if you can spot morphological and ecological differences between these phylogenetic species.

A handful of studies have documented morphologically indistinguishable, cryptic species diversity, for instance, in the fleshy fungus *Sparassis crispa* (Hughes et al. 2014), the polypore genus *Heterobasidion* (Otrosina and Garbelotto 2010) and the corticioid genera *Coniophora* (Kauserud et al. 2007), *Peniophorella* (Hallenberg et al. 2007) and *Sebacina* (Riess et al. 2013). However, there is no clear distinction when a species is morphologically cryptic. In our own experience, while *Antrodiella* spp. and *Skeletocutis* spp. may be identifiable to a few experts with the aid of a phase-contrast microscope, they are cryptic to many mycologists without access to material for comparison (Miettinen and Niemelä 2018, Miettinen et al. 2006). To us, it would appear that truly cryptic species are a small minority in polypores and corticioid fungi—morphological differences can be detected even between closely related species.

Morphological resolution is of significance when we need to link old names and unsequenced historical specimens to modern species concepts. In these cases, the question about morphologically indistinguishable, cryptic species becomes relevant.

Here, we demonstrate previously unrecognised cryptic diversity in a polypore species complex comprising the morphospecies *Skeletocutis nivea* (Jungh.) Jean Keller and *S. ochroalba* Niemelä in the family Incrustoporiaceae Jülich (Polyporales, Basidiomycota). Despite in-depth morphometrics, extensive material and the best of our expertise, we have not been able to find reliable morphological differences between most species in this complex. Species identification is thus reliant on DNA markers only and we have taken the necessary step to provide sequenced types for all known taxa whenever possible.

According to the prevailing, morphology-based circumscription, *S. nivea* is a cosmopolitan white-rot polypore species, distributed in tropical and temperate zones on both hemispheres, growing on dead angiosperm wood. *S. nivea* was originally described from the Island of Java (Indonesia) (Junghuhn 1838) and subsequently basionyms *Polyporus semipileatus* Peck from North America and *Poria coprosmae* G. Cunn. from New Zealand have been widely accepted as synonyms of *S. nivea*. A species currently known as *S. diluta* (Rajchenb.) A. David & Rajchenb. was originally described as a subspecies of *S. nivea* but ITS sequences have shown it to be distinct from *S. nivea* and more closely related to other *Skeletocutis* species (Vlasák et al. 2011).

*S. ochroalba*, described from boreal North America (Niemelä 1985), is very similar to *S. nivea* but grows on coniferous wood. Subsequent findings, identified as this species, have been reported mainly from Eurasia. The morphospecies *S. nivea* and *S. ochroalba* are distinguished from other species of *Skeletocutis* Kotl. & Pouzar by their hyphal structure, which is usually characterised as trimitic in context and monomitic in trama and the very small size of pores and basidiospores. We use the collective term *S. nivea* complex to refer to both morphospecies.

In this study, we resolve the diversity within the *S. nivea* complex by phylogenetic analyses of two genetic markers, viz. the nuclear ribosomal internal transcribed spacer (ITS) region and the translation elongation factor EF-1α (*tef1*), complemented by microscopic study of specimens. We have sampled *S. nivea* and *S. ochroalba* widely over their known distributions and combine our newly generated data with previously published sequences. Based on our results, we propose a taxonomic revision of the *S. nivea* complex.

## Materials and methods

### DNA extraction, amplification and sequencing

In total, 92 ITS and 33 *tef1* DNA sequences were generated for this study. In addition, we included sequences publicly available in the International Nucleotide Sequence Database Collaboration database (INSDC). To elucidate the relationship of the *S. nivea* complex to other taxa in the family Incrustoporiaceae, we also amplified the nuclear large subunit (LSU) region of the rRNA operon from representatives of the *S. nivea* complex and retrieved LSU sequences of relevant outgroup taxa from the INSDC. LSU is highly conserved within the *S. nivea* complex but enables comparisons to more distantly related taxa. All of the newly generated sequences were deposited in the INSDC. Voucher data for all included sequences are provided in Suppl. material 1.

DNA was extracted from dried herbarium samples of basidiocarps and mycelia from agar cultures using the E.Z.N.A. Forensic DNA kit (Omega Bio-tek). Pieces of the sample were cut out with a scalpel and then homogenised with a mortar and pestle in a 1.5 ml centrifuge tube. Further steps were performed according to the kit manufacturer’s protocol.

The primers ITS5 and ITS4 (White et al. 1990) were used for amplifying and sequencing the ITS1–5.8S–ITS2 region of the nuclear rRNA operon. The primers EF1-983.2f (5’-GCH YCH GGN CAY CGT GAY TTY AT-3’) (modified from Matheny et al. 2007) and EF1-G2r (5’-GCD ATR TGN GCR GTR TGR CAR TC-3’) (modified from Rehner 2001) were used for the *tef1*. The LSU region was amplified with the primers CTB6 (Haynes et al. 1995) and LR7 (Vilgalys and Hester 1990) and sequenced with the primers CTB6-LR5 (Vilgalys and Hester 1990) and LR3R (5’-GTC TTG AAA CAC GGA CC-3’)-LR7.

Polymerase chain reactions (PCRs) were carried out with either the Illustra PureTaq Ready-To-Go PCR Beads (GE Healthcare), DreamTaq Green PCR mix (Thermo Scientific) or Phire Tissue Direct PCR Master Mix (Thermo Scientific). A touchdown style PCR programme (designed by Zheng Wang http://wordpress.clarku.edu/polypeet/datasets/primer-information/) was applied for *tef1* amplification. The resulting products were sequenced with BigDye v.3.1 and ABI3730XL analyser (Applied Biosystems) by Macrogen and FIMM. The electropherograms of forward and reverse sequences were aligned against each other using Sequencher v. 5.0 (Gene Codes Corporation). The aligned electropherograms were then visually inspected to ensure good sequence quality and ambiguous sequence reads were discarded. Double peaks were interpreted as true base ambiguities when they were detected in both forward and reverse sequencing electropherograms.

### Alignments and phylogenetic analysis

For an outgroup analysis, a combined ITS–LSU dataset was assembled with representatives of the *S. nivea* complex (18 sequences) and outgroups (14 sequences from 11 species). The resulting trees were rooted with *Tyromyces merulinus* (a possible sister to Incrustoporiaceae) following Justo et al. (2017). The *Tef1* (34 sequences) and ITS (139 sequences) datasets were analysed to investigate the phylogeny of the *S. nivea* complex in more detail and to delineate species within the complex. *Tyromyces globosporus* (JN710734) was used as an outgroup in the *tef1* analysis and *Skeletocutis chrysella* (JQ673126) in the ITS analysis.

Sequences were aligned using PRANK v.140603 (Löytynoja and Goldman 2010) with default settings. These alignments were checked by eye and alignment errors were corrected manually. In the ITS-LSU analysis, parts of the ITS region were so divergent between distantly related taxa that they could not be credibly aligned and those sections (131/706, 18.6% of aligned positions) were excluded from the ITS–LSU analysis. The alignments and trees were deposited in TreeBASE (22552).

Phylogenetic trees were constructed using the maximum likelihood (ML) and Bayesian inference (BI) methods. ML trees were reconstructed with RAxML 8.2.10 (Stamatakis 2014) with GTR+G set as the evolutionary model and alignments partitioned according to Table 1. The rapid bootstrap with MRE-based bootstopping criterion option was used for branch support estimation.

**Table 1.** Sequence partitions and substitution models.

Analysis	Gene	Partition in model estimation	Model selected for Bayesian analysis	Sub-partition	Length in alignment	Number of parsimony informative sites in alignment
outgroup	nrRNA	ITS1 and ITS2	GTR+G	ITS1	215	62
				ITS2	206	67
		5.8S and LSU	K80+G	5.8S	154	8
				LSU	1460	91
ingroup		ITS1 and ITS2	HKY+G	ITS1	257	57
				ITS2	262	73
		5.8S	K80		154	0
	*tef1*	1. codon position	F81+G		231	13
		2. codon position	JC+G		231	11
		3. codon position	HKY+G		231	87
		introns	HKY+I		188	79

The substitution models for BI were selected for each partition (Table 1) with JModelTest2 v.2.1.6 (Guindon and Gascuel 2003, Darriba et al. 2012) prior to the run. When evaluating models, the number of substitution schemes was set to 3 and models with equal/unequal base frequencies (+F), with/without a proportion of invariable sites (+I) and with/without rate variation amongst sites (+G) (nCat = 25), were included. Models were selected according to Bayesian information criterion.

BI analyses were performed in MrBayes v.3.2.6 (Ronquist et al. 2012) in two independent runs with 4 chains for 10 million generations for the analysis of ITS sequences and 1 million generations for *tef1* and ITS–LSU analyses. Upon run completion, the first 25% of the trees were discarded as burn-in. By then, the average standard deviation of split frequencies had reached a value lower than 0.01.

All analyses were performed through the Cipres Science Gateway v.3.3 interface (Miller et al. 2010).

The numbers of base substitutions per site within and between the inferred species were calculated in MEGA v.7.0.25 (Kumar et al. 2016) by averaging over all sequence pairs in an analysis with Maximum Composite Likelihood model (Tamura et al. 2004). The rate variation amongst sites was modelled with a gamma distribution (shape parameter = 4). The differences in the composition bias amongst sequences were considered in evolutionary comparisons (Tamura and Kumar 2002). Standard error estimates were obtained by a bootstrap procedure (500 replicates).

### Microscopic study

The majority of studied materials are dried specimen collections stored in herbarium H (Helsinki). Type material and reference specimens from herbaria BPI, H, L, O and PDD were also studied. Herbarium acronyms are given according to Thiers (2017).

Pore measurements (12 per specimen) were done under a stereomicroscope (Wild M54) by counting the number of pores per 1 mm; only pores aligned in straight rows were selected for this purpose. Microscopic structures were studied and measured with Leitz Diaplan and Leica DMBL microscopes (×1250 magnification). Microscopic routines used in this study follow Miettinen et al. (2006, 2012). Measurements were made and illustrations were drawn in Cotton Blue using phase contrast illumination and oil immersion (with a subjective accuracy of 0.1 μm; Miettinen et al. 2006).

In microscopic descriptions, the following abbreviations are used: L – mean spore length; W – mean spore width; Q – mean L/W ratio; n – pore counts, spores or hyphae measured / number of specimens. For presenting a variation of basidiospores and hyphae, 5% of measurements were excluded from each end of the range and are given in parentheses. The respective cut-off for reported pore measures is 20%.

## Results

### Sequences and phylogeny

Intragenomic variation of ITS sequences, as discussed by Lindner and Banik (2011) and Lindner et al. 2013, was found to be common and widespread in the *S. nivea* complex. Variation consisted mainly of single nucleotide polymorphisms (SNP) and short length variations of one or a few base pairs. In our analyses and INSDC submission, we always used the shortest alleles.

**Figure 1.**
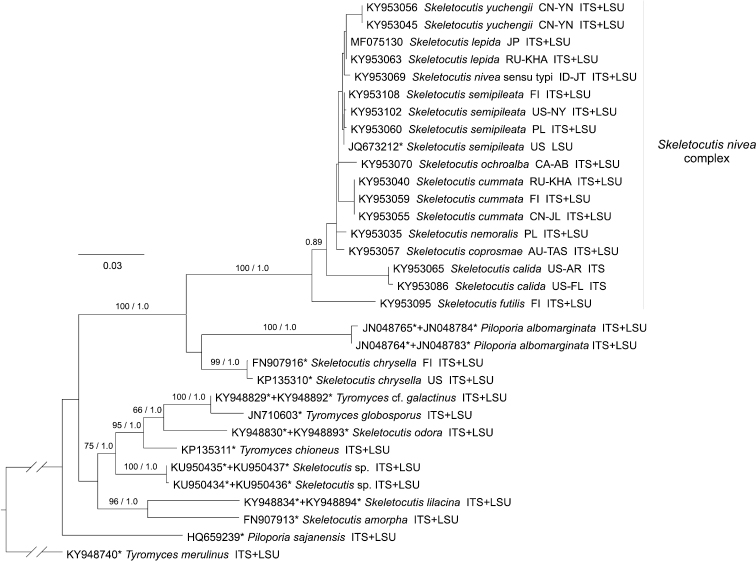
Phylogenetic tree from ML analysis of the ITS–LSU dataset. Bootstrap support values (up to 100) and respective Bayesian posterior probabilities (up to 1) are shown beside branches (bs / pp) where bs > 50 or pp ≥0.80. Terminal labels include INSDC accession number(s), species name, (area of origin, in ISO 3166 code) and gene regions included. * = sequence retrieved from the INSDC.

Phylogenetic analyses of the ITS–LSU dataset (Fig. 1) show that the *S. nivea* complex forms a monophyletic assemblage of closely related species in the family Incrustoporiaceae. The genus *Skeletocutis* in its current circumscription is non-monophyletic and the *S. nivea* complex belongs to a clade that is separate from the type species of the genus, *S. amorpha* (Fr.) Kotl. & Pouzar. When the generic limits within the family are clarified in the future, the *S. nivea* complex should be assigned to a different genus.

Some segments of ITS sequences proved difficult to align unequivocally even within the *S. nivea* complex. While the composition of the clades which we interpret as species was not affected, the topologies of deeper nodes were found to be somewhat sensitive to small adjustments of the alignment in those variable segments.

**Figure 2.**
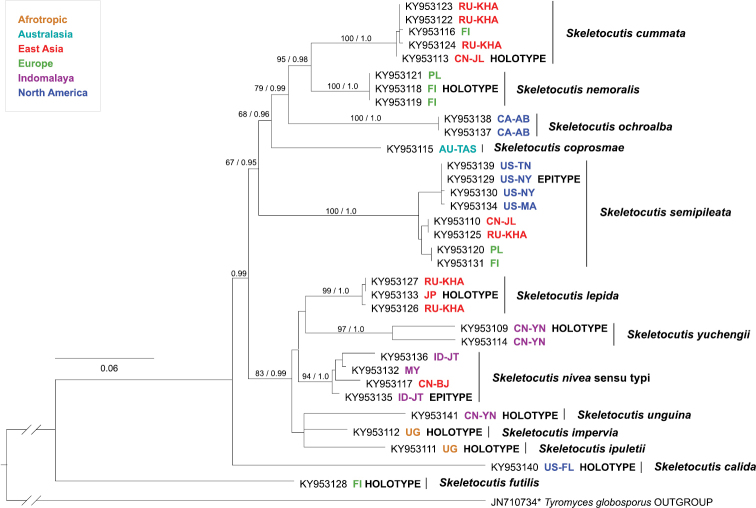
Phylogenetic tree from ML analysis of the *tef1* dataset. Bootstrap support values (up to 100) and respective Bayesian posterior probabilities (up to 1) are shown beside branches (bs / pp) for all nodes that delimit species and for deeper nodes where bs >50 or pp ≥0.95. Terminal labels include INSDC accession number, species name, area of origin (in ISO 3166 code) and indication of type status. * = sequence retrieved from the INSDC.

**Figure 3.**
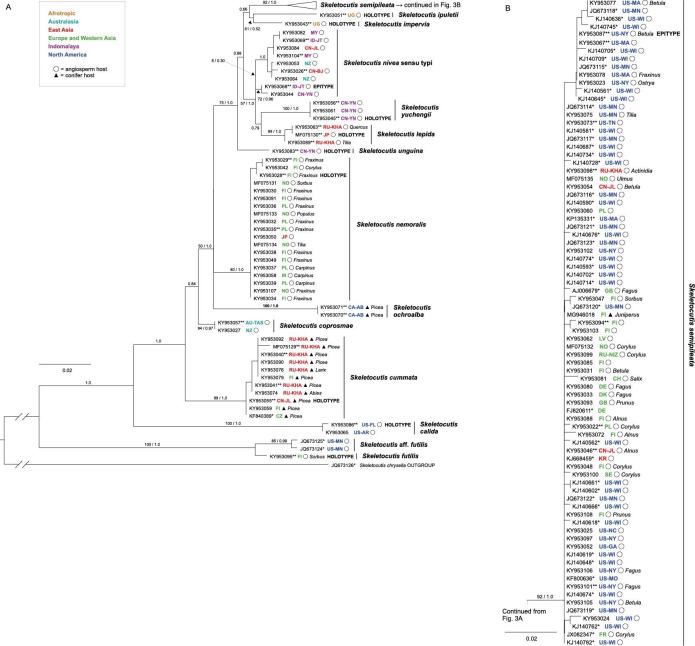
Phylogenetic tree from ML analysis of the ITS dataset. Bootstrap support values (up to 100) and Bayesian posterior probabilities (up to 1) are shown beside branches (bs / pp) for all nodes that delimit species and deeper nodes where bs >50 or pp ≥0.95. Terminal labels include INSDC accession number, species name, area of origin (in ISO 3166 code), host tree and indication of type status. * = sequence retrieved from the INSDC. ** = *tef1* sampled from corresponding specimen. (A) All terminals shown except *Skeletocutis semipileata*; (B) terminals within *S. semipileata.*

Despite partially contrasting topology of inter-group relationships, the phylogenetic analyses of both *tef1* (Fig. 2) and ITS (Fig. 3) datasets were concordant in grouping terminals into 13 clades that we interpret and describe here as species. Analysis of the ITS dataset revealed an additional 14^th^ clade (S. aff. futilis) not included in our *tef1* sampling, which may represent a separate species.

All recognised species were strongly supported in the analyses of the *tef1* dataset. Corresponding support from the ITS data was strong for all but *S. nivea*, which lacks true synapomorphic characters in relation to *S. lepida* and *S. yuchengii* in that genetic marker.

Average estimated intraspecific sequence divergence in the ITS dataset was up to 0.32% (SE=0.12) (in *S. nivea*) and in the *tef1* dataset up to 4.2% (SE=0.8) (in *S. yuchengii*). Average estimated genetic distances between species varied from 1.3% (SE=0.4) between *S. lepida* and *S. nivea*, to 10.3% (SE=1.1) between *S. calida* and S. aff. futilis in the ITS dataset and from 3.7% (SE=0.7) between *S. impervia* and *S. lepida*, to 16.9% (SE=2.1) between *S. futilis* and *S. ochroalba* in the *tef1* dataset. The genetic divergence within and between species was generally higher in *tef1* than ITS sequences. The full set of estimates of genetic divergence between and within species is provided in Suppl. materials 2–4.

### Morphology, ecology and distribution

Morphometrics for each species are reported in Table 2. Characteristics and ecology of the species are summarised in Table 3. Distribution maps depicting the approximate collection localities of specimens are provided in Supplement S5A–C.

**Table 2.** Pore and spore measurements of species in the *Skeletocutis nivea* complex. Spore measures in bold-face are accumulated statistics from specimens below.

Specimen	Pores/mm (min–median–max)	n	Spore length (µm)	L	Spore width (µm)	W	Q’	Q	n
***Skeletocutis calida* sp. nov.**			**2.5–3.1(–3.3)**	**2.86**	**0.5–0.6(–0.7)**	**0.55**	**(4.0–)4.3–6.0(–6.9)**	**5.18**	**60/2**
holotype	8–**9**–10	12	2.8–3.0	2.92	0.5–0.6(–0.7)	0.56	(4.3–)4.8–5.8(–6.0)	5.22	30
Miettinen 17466	8–**9**–11	12	2.5–3.2(–3.3)	2.79	0.5–0.7	0.54	(4.0–)4.2–6.4(–6.9)	5.13	30
***S. coprosmae* comb. nov.**			**2.8–3.2(–3.3)**	**2.98**	**0.5–0.7**	**0.57**	**(4.0–)4.3–6.0(–6.4)**	**5.19**	**60/2**
holotype	6–**8**–9	12	2.8–3.2(–3.3)	3	0.5–0.7	0.57	4.3–6.2(–6.4)	5.3	30
Gates 1898	7–**8**–9	12	2.8–3.1	2.95	0.5–0.7	0.58	(4.0–)4.3–6.0	5.09	30
***S. cummata* sp. nov.**			**(2.8–)2.9–3.4(–3.9)**	**3.1**	**0.5–0.8(–0.9)**	**0.66**	**(3.3–)3.8–6.0(–6.6)**	**4.68**	**270/9**
holotype	5–**6**–7	12	2.9–3.3	3.05	0.6–0.8(–0.9)	0.74	(3.3–)3.6–5.2(–5.5)	4.14	30
Niemelä 9088	6–**7**–8	12	3.0–3.3(–3.5)	3.09	0.6–0.7	0.66	4.3–5.3	4.71	30
Spirin 3857	7–**8**–9	12	(2.9–)3.0–3.9	3.13	0.5–0.8	0.63	4.3–6.0	4.94	30
Spirin 4170	7–**9**	12	2.9–3.7(–3.9)	3.18	0.5–0.7(–0.8)	0.58	4.4–6.4(–6.6)	5.48	30
Spirin 4897	6–**7**–8	12	2.8–3.4(–3.5)	3.05	0.5–0.7	0.6	(4.1–)4.3–6.0	5.11	30
Spirin 5430	5–**6**–8	12	3.0–3.4	3.1	0.5–0.8	0.66	(3.9–)4.0–6.0	4.68	30
Spirin 5472	6–**7.5**–9	12	3.0–3.4(–3.5)	3.09	0.6–0.8(–0.9)	0.74	3.8–5.0	4.18	30
Spirin 5484	7–**10**–13	12	2.9–3.2(–3.3)	3.05	0.6–0.8	0.67	3.6–5.2(–5.3)	4.52	30
Spirin 5676	7–9	12	2.8–3.4(–3.5)	3.12	0.6–0.8	0.67	(3.5–)3.9–5.7	4.63	30
***S. futilis* sp. nov.**	6–**7**–8	9	3.0–4.0	3.33	0.7–0.9	0.81	(3.3–)3.4–5.1	4.13	30
***S. impervia* sp. nov.**	7–**8.5**–10	12	(2.8–)2.9–3.1	2.97	0.5–0.8	0.61	(3.6–)3.8–6.0(–6.2)	4.85	30
***S. ipuletii* sp. nov.**	9–**10**–11	12	2.8–3.4	2.96	0.5–0.7(–0.8)	0.6	(4.0–)4.1–6.0(–6.2)	4.97	30
***S. lepida* sp. nov.**			**(2.8–)2.9–3.0(–3.1)**	**2.95**	**0.5–0.6**	**0.55**	**4.8–6.0**	**5.36**	**90/3**
holotype	7–**8**–9	12	2.9–3.1	2.98	0.5–0.6	0.54	4.8–6.0	5.55	30
Spirin 4989	7–**9**–10	12	(2.8–)2.9–3.0	2.94	0.5–0.6	0.56	4.8–6.0	5.22	30
Spirin 3964	7–**9**–10	12	2.8–3.0	2.93	0.5–0.6	0.55	4.8–6.0	5.3	30
***S. nemoralis* sp. nov.**			**(2.8–)2.9–3.2(–4.0)**	**3.04**	**(0.4–)0.5–0.6(–0.7)**	**0.56**	**(4.1–)4.8–6.3(–7.8)**	**5.47**	**390/13**
holotype	6–**7.5**–9	12	2.9–3.2(–3.3)	3.06	0.5–0.7	0.57	4.3–6.2	5.4	30
Brandrud 149–04	7–**8**–9	12	(2.9–)3.0–3.4(–3.8)	3.13	0.4–0.7	0.55	(4.6–)4.9–7.2(–7.5)	5.66	30
Gaarder 5257	8–**8.5**–9	12	(2.9–)3.0–3.8(–4.0)	3.17	(0.4–)0.5–0.7	0.57	(4.6–)4.7–6.7(–7.5)	5.59	30
Klepsland JK06–S080	7–**8**–9	12	2.8–3.0(–3.2)	2.94	0.5–0.7	0.57	4.1–6.0	5.16	30
Korhonen 28	7–**8**–9	12	2.9–3.1(–3.3)	3.02	0.4–0.6(–0.7)	0.54	(4.7–)4.8–7.2(–7.8)	5.56	30
Korhonen 31	6–**7**–9	12	2.9–3.1(–3.5)	3.02	0.5–0.7	0.58	4.3–6.0(–6.2)	5.18	30
Korhonen 35	7–7–8	12	(2.8–)2.9–3.2(–3.3)	3.06	0.5–0.6	0.54	(4.8–)5.0–6.4(–6.6)	5.71	30
Korhonen 83	6–**8**–9	12	2.9–3.1	3	0.5–0.6(–0.7)	0.55	(4.3–)4.8–6.2	5.46	30
Korhonen 86	7–**8**–10	12	2.9–3.1	3	0.5–0.6	0.57	4.8–6.0(–6.2)	5.3	30
Korhonen 89	7–**8**–9	12	2.9–3.1(–3.2)	3.05	0.5–0.6	0.56	4.8–6.2	5.45	30
Korhonen 93	6–**7**–8	12	3.0–3.1	3.05	0.4–0.6	0.53	5.0–7.5(–7.8)	5.79	30
Korhonen 100	7–**8**	12	2.9–3.1(–3.2)	3.02	0.5–0.6	0.56	4.8–6.2	5.43	30
Korhonen 103	6–**7**–8	12	(2.8–)2.9–3.2(–3.8)	3.04	(0.4–)0.5–0.6	0.55	(4.7–)4.8–6.4(–7.2)	5.52	30
***S. nivea***			**(2.7–)2.8–3.2(–3.7)**	**2.96**	**0.5–0.7(–0.8)**	**0.56**	**(3.9–)4.3–6.0(–6.2)**	**5.27**	**125/5**
holotype	8–**10**–13	5	–	–	–	–	–	–	–
epitype	8–**10**–11	12	(2.7–)2.8–3.4(–3.7)	2.96	0.5–0.7(–0.8)	0.56	(4.3–)4.4–6.0	5.32	30
Miettinen 10579.1	7–**8**–9	12	2.7–3.0	2.91	0.5–0.7	0.57	(3.9–)4.0–6.0	5.08	30
Miettinen 18255	–	–	3.0–3.2	3.12	0.5–0.6	0.56	5.2–6.2	5.57	5
Miettinen 16350	9–**10**–11	12	2.7–3.1	2.91	0.5–0.7	0.55	4.3–6.2	5.33	30
Ryvarden 38177	7–**8**–10	12	2.8–3.5(–3.7)	3.02	0.5–0.7	0.57	(4.1–)4.3–6.0	5.3	30
***S. ochroalba***			**(2.8–)2.9–3.7(–4.0)**	**3.1**	**0.5–0.8**	**0.67**	**3.8–6.0(–7.0)**	**4.65**	**70/3**
Niemelä 2689	6–**7.5**–9	12	2.9–3.7(–4.0)	3.1	(0.5–)0.6–0.8	0.67	(3.8–)4.1–5.2(–7.0)	4.63	35
Spirin 8854a	6–**7**–9	22	(2.8–)2.9–3.2(–3.3)	3.04	0.5–0.8	0.67	3.8–6.0(–6.2)	4.56	30
Spirin 8854b	7–8–10	9	3.2–3.8	3.48	0.6–0.7	0.66	4.7–5.5	5.27	5
***S. semipileata* comb. nov.**			**(2.3–)2.8–3.1(–3.3)**	**2.97**	**0.4–0.6(–0.7)**	**0.55**	**(4.1–)4.7–7.0(–7.5)**	**5.43**	**450/15**
lectotype	8–**8.5**–11	10	–	–	–	–	–	–	–
epitype	7–**8**–10	12	2.8–3.1	2.98	0.5–0.7	0.57	4.4–6.0	5.22	30
Gaarder 5136	7–**8**–10	12	(2.8–)2.9–3.1(–3.3)	2.98	0.5–0.7	0.58	(4.1–)4.4–6.0	5.17	30
Korhonen 76	7–**9**–10	12	(2.6–)2.8–3.1	2.98	0.4–0.6(–0.7)	0.53	(4.1–)4.8–7.2	5.62	30
Miettinen 6694	7–**8**–10	12	(2.7–)2.8–3.2	2.97	0.5–0.6	0.58	4.8–6.0	5.16	30
Miettinen 14114	7–**8**–10	12	2.8–3.0(–3.1)	2.94	0.5–0.6	0.56	4.8–6.0	5.28	30
Miettinen 14917.4	8–**9**–10	12	(2.8–)2.9–3.1	3	(0.4–)0.5–0.6	0.55	(4.7–)4.8–6.2(–7.5)	5.42	30
Miettinen 15715	8–8–9	12	(2.3–)2.4–3.0	2.79	(0.4–)0.5–0.6	0.53	(4.5–)4.6–5.8(–7.0)	5.26	30
Miettinen 15835	8–**9**–11	12	(2.8–)2.9–3.2(–3.3)	3.02	(0.4–)0.5–0.6	0.54	5.0–6.2(–7.0)	5.56	30
Miettinen 16693.1	8–8–9	12	2.9–3.2(–3.3)	3.01	0.4–0.6(–0.7)	0.52	(4.3–)4.8–7.5	5.75	30
Miettinen 16823	8–**9**–11	12	2.8–3.0	2.93	0.4–0.6	0.49	4.8–7.5	5.93	30
Miettinen 17074	8–**9**–11	12	2.8–3.0	2.93	0.4–0.7	0.56	(4.1–)4.3–7.2	5.27	30
Miettinen 17135	7–**9**–10	12	2.9–3.0(–3.1)	2.99	(0.4–)0.5–0.6	0.54	5.0–6.0(–7.5)	5.5	30
Ryvarden 47279	7–**8**–10	12	2.9–3.1	3.02	0.5–0.7	0.58	(4.3–)4.4–6.0(–6.2)	5.21	30
Spirin 2326	7–**8**–9	12	2.8–3.1	2.96	0.4–0.6	0.53	(4.7–)4.8–7.0(–7.2)	5.59	30
Spirin 5142	8–8–10	12	2.9–3.0(–3.1)	2.98	0.4–0.6	0.53	(4.8–)5.0–7.2	5.59	30
***S. unguina* sp. nov.**	7–**8**–9	12	2.9–3.2(–3.3)	3.04	(0.4–)0.5–0.6(–0.7)	0.55	(4.6–)4.8–6.4(–7.5)	5.49	30
***S. yuchengii* sp. nov.**			**(2.7–)2.8–3.1(–3.2)**	**2.96**	**(0.4–)0.5–0.7**	**0.59**	**(4.0–)4.1–6.0(–7.2)**	**4.99**	**90/3**
holotype	8–**8.5**–11	12	(2.8–)2.9–3.2	3.01	0.5–0.7	0.62	(4.0–)4.1–6.0(–6.4)	4.86	30
Miettinen 10150.2	–	–	2.7–3.1	2.93	0.5–0.7	0.59	(4.0–)4.1–6.0(–6.2)	5	30
Miettinen 10366.1	8–**9**–11	12	(2.8–)2.9–3.0(–3.1)	2.95	(0.4–)0.5–0.7	0.58	(4.0–)4.1–6.0(–7.2)	5.11	30

**Table 3.** Distribution, ecology and habit of species in the *Skeletocutis nivea* complex.

Host	Species	Distribution	Characteristics and ecology
angiosperm	*Skeletocutis calida* sp. nov.	subtropical N America	annual; basidiocarps small individual pilei; on fallen twigs
	*S. coprosmae* comb. nov.	Tasmania, New Zealand	**potentially perennial**; basidiocarps becoming large and sturdy when growing on coarse wood
	*S. futilis* sp. nov.	Finland	annual; basidiocarps small; **spores over 0.7 µm thick**; on fallen twigs
	*S. impervia* sp. nov.	Uganda	likely annual
	*S. ipuletii* sp. nov.	Uganda	likely annual
	*S. lepida* sp. nov.	temperate East Asia (Russian Far East, Japan)	annual; basidiocarps small individual pilei when growing on thin branches, sturdier on coarse wood
	*S. nemoralis* sp. nov.	temperate Eurasia	annual; basidiocarps often sturdy and large; on fallen branches; **prefers *Fraxinus***
	*S. nivea*	China, SE Asia, New Zealand	annual; basidiocarps potentially quite large; on fallen branches or logs
	*S. semipileata* comb. nov.	temperate – south-boreal northern hemisphere	annual; basidiocarps often quite large; on fallen twigs, branches or logs of various woody angiosperms
	*S. unguina* sp. nov.	China (Yunnan)	annual; basidiocarps small individual pilei; on fallen twigs
	*S. yuchengii* sp. nov.	China (Yunnan)	annual; basidiocarps small; on fallen twigs
conifer	*S. cummata* sp. nov.	(oro)boreal Eurasia	annual; basidiocarps small, pileus surface and margin slightly pubescent; on downed conifer logs, usually *Picea*
	*S. ochroalba*	boreal N America	potentially perennial; basidiocarps small, pileus surface and margin slightly pubescent; on downed logs of *Picea*

## Discussion

### Phylogeny and species diversity in the *Skeletocutis nivea* complex

Interspecies relationships were not clearly resolved by our data and analyses of the ITS and *tef1* datasets resulted in partially contrasting topologies. *Skeletocutis futilis* (together with S. aff. futilis) and *S. calida* represent long, divergent branches which are consistently positioned as early diverging lineages in our analyses. However, the position of *S. calida* was found to be sensitive to slight alterations of alignment in some variable segments of ITS, alternative positions being within the crown group.

The crown group is characterised by relatively short internodes and poor interspecies resolution. However, *S. lepida*, *S. nivea* and *S. yuchengii* were consistently grouped together. Weak support (bs=9, pp=0.30) for *S. nivea* in the ITS data could be explained by incomplete lineage sorting in this genetic marker after relatively recent speciation. The loss of ancestral alleles can be expected to be slower in the widely distributed *S. nivea*, which probably comprises a larger population than the regionally endemic *S. lepida* or *S. yuchengii*.

Despite their ecological and morphological similarities, the conifer-dwelling species *S. cummata* and *S. ochroalba* were recovered as sister species only in the Bayesian analysis of the ITS–LSU dataset. Even then, the support was minimal (pp=0.151).

Our results with the *S. nivea* complex are in line with other molecular systematic studies in *Polyporales* (e.g. Carlson et al. 2014, Miettinen et al. 2018) who conclude that *tef1* is more variable between species than ITS and provides greater resolution in separating species. Still, unique character state combinations can be identified from ITS sequences even for the most closely related species in the *S. nivea* complex. Intraspecific sequence variation in *tef1* was generally larger but roughly corresponding to the variation observed in the ITS region. However, in *S. yuchengii*, the *tef1* sequences show striking divergence in contrast to ITS. To clarify interspecies relationships within the *S. nivea* complex, additional genetic markers such as the *rpb1* will need to be sequenced.

### Geographic patterns of diversity

Our sampling is concentrated in the northern temperate zone, where most species of the *S. nivea* complex appear to be restricted to a single continent or region. This is in accordance with other comparable studies on polypores (e.g. Otrosina and Garbelotto 2010, Spirin et al. 2015, Han et al. 2016, Song and Cui 2017, Spirin et al. 2017, Miettinen et al. 2018). Notable exceptions here include *S. semipileata*, which has a circumpolar distribution and *S. nivea*, which ranges longitudinally from northern China to New Zealand.

The greatest species diversity is found in Eurasia and particularly in East Asia with three species unique to the region: *S. lepida* in Northeast Asia and *S. unguina* and *S. yuchengii* in southern China. *S. futilis* is thus far known only from northern Europe but the closely related S. aff. futilis occurs in North America. *S. cummata* and *S. nemoralis* have continent-wide distributions in Eurasia.

North American species include *S. calida* in southern U.S.A. and S. aff. futilis in northern U.S.A. The conifer-dwelling *S. ochroalba* represents the North American parallel of the Eurasian *S. cummata* with boreal, continent-wide distribution.

Wide and disjunct distributions of species like *S. semipileata*, *S. nivea* and *S. coprosmae* indicate that species distributions are not necessarily limited by dispersal ability. Spores in the *S. nivea* complex are exceedingly small and their theoretical dispersal ability along air currents is practically unlimited (Wilkinson et al. 2012). Yet, the actual dispersal ability may be severely limited by the survivability of the delicate spores during long distance transport and their ability to establish after deposition on a new substrate (Norros et al. 2015).

The generalist ecology of *S. semipileata* may have facilitated its dispersal over the northern hemisphere, while some of the more restricted species may be limited by low establishment probability imposed by stricter specialisation. Geographic structuring within *S. semipileata*, particularly evident in the *tef1* data – where differentiation between North America and Eurasia and furthermore East Asian and European populations emerges – suggests that gene flow across long distances in this species is somewhat restricted. Similarly, geographic isolation is likely driving the differentiation between *S. futilis* and S. aff. futilis, be that inter or intraspecific.

Our sampling from the low latitudes and southern hemisphere was sporadic but yielded a proportionately large number of species, most of which were represented by only one or a few specimens. Specimens collected from a relatively small area in Yunnan, southern China fall into three species, two of which (*S. unguina* and *S. yuchengii*) are known only from that area.

The only two African specimens, both from Uganda, proved to represent separate species, *S. impervia* and *S. ipuletii*. Another new species, *S. afronivea* Ryvarden, morphologically close to the *S. nivea* complex, was recently described from Uganda (Ryvarden 2018). Sequence data for *S. afronivea* is not available, so its affinity to the *S. nivea* complex cannot be verified. However, the larger spore size distinguishes *S. afronivea* as separate from *S. impervia*, *S. ipuletii* and most other species of the *S. nivea* complex.

We anticipate that further studies are likely to reveal even more diversity within the *S. nivea* complex. Potential hotspot areas include the montane forests of the tropics as well as the temperate forests in mid-latitudes. For instance, Western North America and large parts of the southern hemisphere were not sampled in this study.

### Ecology and host tree associations

Ecologically, *S. nivea* complex can be divided into conifer-dwelling species (*S. cummata* and *S. ochroalba*) and angiosperm-dwelling species. The lack of support for a sister species relationship between *S. cummata* and *S. ochroalba* suggests that host switching may have happened more than once during the diversification of the *S. nivea* complex. Both *S. cummata* and *S. ochroalba* have remarkably similar fruiting body morphology characterised by small size, pileate form with pubescent pileus surface and the occasional salmon colour on the pore surface as well as spore dimensions that are distinct from most angiosperm-dwelling species. Better phylogenetic resolution of the *S. nivea* complex would be required to discern whether these shared traits represent homologies or independently derived ecological adaptations.

The host range of the conifer-dwelling species remains unresolved. They are most commonly found on logs of *Picea*, but *S. cummata* in the Russian Far East has been collected from *Abies* and *Larix* as well. We also studied a specimen from China (L. Ryvarden 21394 (H)), labelled as *S. nivea* from *Pinus*, but close inspection revealed the spores to be too small for *S. cummata*. The specimen was not sequenced so its true species identity remains unverified. In North America, Overholts (1953) and Lowe (1966) report *S. semipileata* (*P. semipileatus*) as sometimes growing on conifers such as *Picea*, *Pinus* and *Thuja*. It is possible that the referenced specimens belong to *S. ochroalba*, but we have not confirmed their identity. However, we have confirmed one Finnish collection of *S. semipileata* (O. Miettinen 21003 (H)) from *Juniperus communis*, proving that occasional crossovers of angiosperm-dwelling species to conifer hosts sometimes occur.

Several of the angiosperm-dwelling species are so far known only from one or a few specimens and detailed substrate data were often not available. Our records of *S. lepida*, *S. nemoralis* and *S. semipileata* suggest that individual species are able grow on a wide diversity of woody angiosperms. A preference for *Fraxinus* wood is evident in *S. nemoralis*, whereas *S. semipileata* appears to be rather indifferent in this respect. All species can be found on thin branches, but some (*S. nivea* and *S. semipileata*) have also been recorded from coarse woody debris (>20 cm Ø). The examples presented above indicate that ecological specialisation amongst the angiosperm-dwelling species is relatively weak. However, generalisations from common and widespread species, such as *S. semipileata*, are likely to be biased. Some degree of niche partitioning could be expected at least locally, where two or more species co-occur.

The observed pattern of overlapping species distributions in the *S. nivea* complex indicates that effective hybridisation barriers are in place between species. The mechanisms of reproductive isolation and the evolutionary processes that have led to their formation are thus far unverified. The existence of possible innate reproductive barriers that prevent hybridisation on shared substrates could be investigated by mating experiments. However, in order to conduct such studies, fresh material will need to be collected to establish living cultures of the species in the *S. nivea* complex. Further ecological study is also required to elucidate possible higher resolution patterns of distribution and substrate use. Special care should be taken to record detailed collection data including the species, size class, quality, position and decay stage (following e.g. Söderström (1988)) of the substrate.

### Crypticity in the *Skeletocutis nivea* complex

After macroscopic and microscopic study of 60 specimens representing 13 species (more than 700 pores, 1700 spores and 3000 hyphae measured), the majority of the species in the *S. nivea* complex are left without reliable morphological diagnosis. Intraspecific variability in basidiocarp phenology appears to be too wide to infer interspecific differences. On the other hand, the microscopic structures of the hyphal system are remarkably uniform across species. Easily measurable quantitative characters such as the spore dimension provide only minimal differentiation, if any. Identification is particularly problematic amongst the angiosperm-dwelling species, many of which clearly have overlapping distributions; only *S.* (aff.) *futilis* is distinguished by distinctly larger spores.

## Taxonomy

A collective description of the *S. nivea* complex is provided below. Individual species descriptions focus on relevant specifications for each species.

### Skeletocutis nivea complex

Taxon classificationFungiPolyporalesPolyporaceae

#### Description.

**Figure 4.**
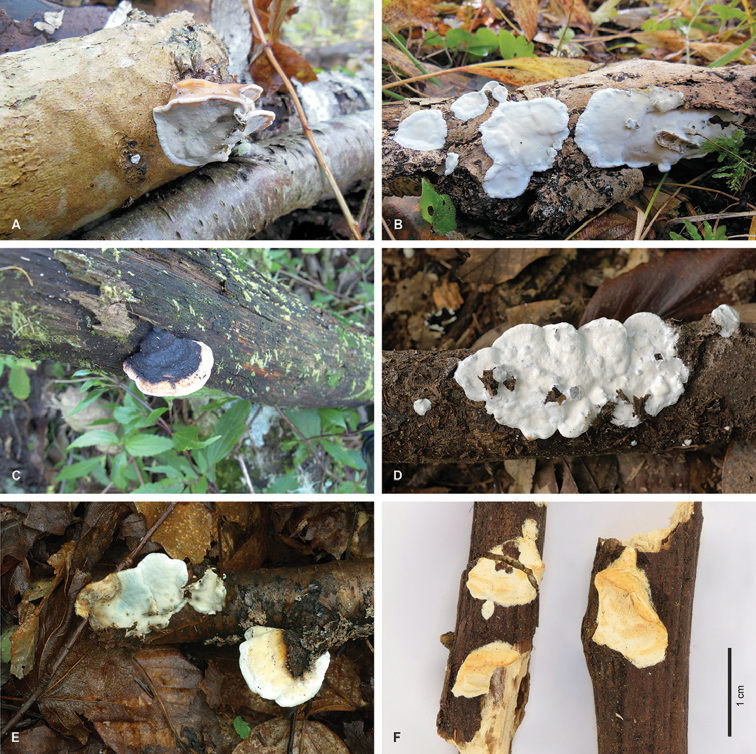
Fruiting bodies of the *Skeletocutis nivea* complex. **A** *S. nemoralis*, Korhonen 86 **B** *S. nemoralis*, Korhonen 89 **C** *S. nivea*, epitype **D** *S. nivea*, Miettinen 16350 **E** *S. semipileata* with a characteristic bluish colour on pore surface, epitype **F** *S. unguina*, holotype.

**Basidiocarps** (Fig. 4) annual to sometimes perennial; half-resupinate (resupinate with a pileate edge) to resupinate; hard when dry; surface of pileus white to ochraceous, sometimes turning black when old (Fig. 4C); pore surface cream coloured with ochraceous tints, bluish or greenish colour sometimes develops in the tubes (Fig. 4E); context and subiculum with coriaceous consistency and whitish colour; pores 6–10 per mm; tube layer darker than context.

**Figure 5.**
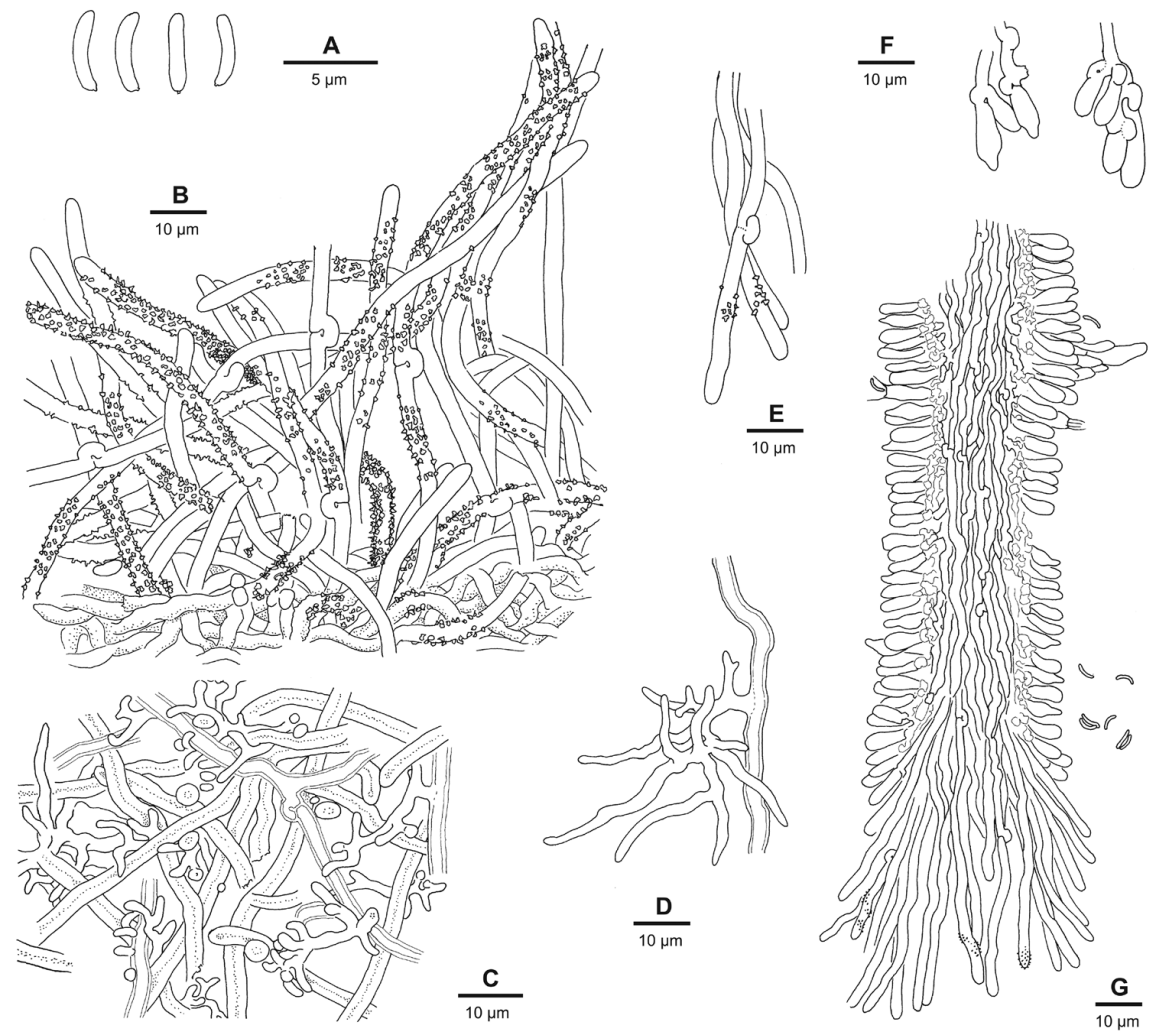
Microscopic structures of *Skeletocutis ochroalba* (reproduced after Niemelä (1985)). **A** spores **B** encrusted tomentum hyphae arising from dense cortical tissue **C** section through context, showing generative and skeletal hyphae and ramified side-branches resembling binding hyphae **D** ramified arbuscule-like binding hypha, arising from a generative hypha **E** dissepiment edge hyphae **F** cystidioles and basidioles **G** vertical section through a dissepiment edge, showing trama, hymenium with a hyphal peg and sparsely encrusted dissepiment edge hyphae.

**Hyphal structure**: context and subiculum seemingly trimitic (Fig. 5C); hyphae are parallel near cap surface, forming a homogenous, coriaceous texture; skeletal hyphae prevailing, unbranched, thick-walled and often solid, refractive; generative hyphae relatively scarce, clamped, sometimes with (unevenly and irregularly) thickened walls and rarely with sandy encrustation, rarely producing generocystidia (encrusted tips of generative hyphae) with thorny encrustation; ‘binding hyphae’ (Fig. 5C–D) 1–2–4 µm wide, arbuscule-like, simple-septate side-branches of generative hyphae, thin-walled to solid and refractive, developing later than skeletal hyphae and sometimes missing in young parts of context/subiculum but becoming dominant in older parts, sometimes filling up the old tube layer.

**Figure 6.**
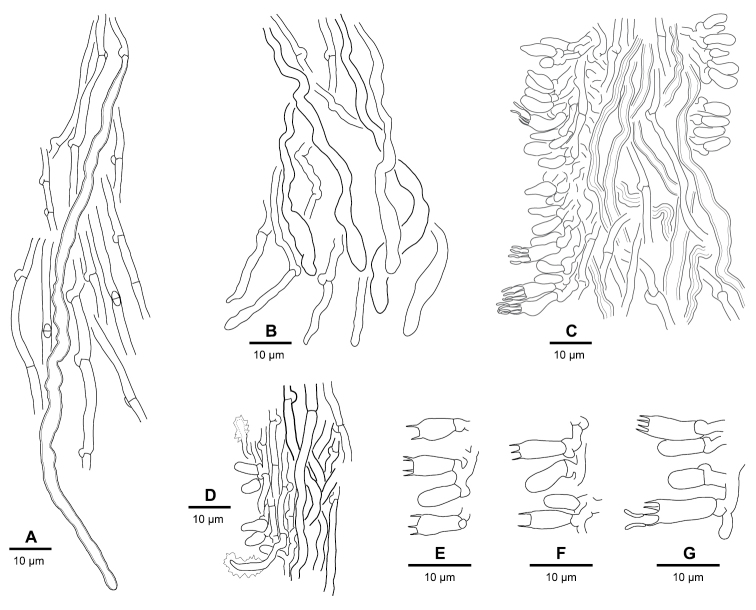
Microscopic structures of the *Skeletocutis nivea* complex. **A** *S. lepida*, tramal skeletal hypha amongst generative hyphae (holotype) **B** *S. semipileata*, ends of generative and skeletal hyphae in trama (Miettinen 17135) **C** *S. nemoralis*, tube trama and hymenium (holotype) **D** *S. nivea*, tube trama and hymenium with encrusted generocystidia (epitype) **E** *S. nivea*, basidia (Miettinen 16350) **F** *S. semipileata*, basidia (epitype) **G** *S. cummata*, the largest basidia in the the *S. nivea* complex (Niemelä 9088).

**Trama** (Fig. 5G) monomitic to dimitic; hyphae interwoven, tightly subparallel; generative hyphae 1–3 µm wide, usually prevailing, clamped, thin-walled or sometimes with slightly thickened walls; skeletal hyphae (Fig. 6A–C) looking different from those in context and subiculum, sparse, sometimes apparently missing, originating from tramal generative hyphae, winding and irregularly wide (up to 5+ µm) with spacious lumen, walls usually only slightly thickened, slightly refractive; generative hyphae in dissepiment edges (Fig. 5E and G) ca. 2 µm wide, thin-walled, slightly undulating, often somewhat irregularly shaped towards the tips, bare to richly encrusted with sandy crystals.

**Hymenium** with fusiform cystidiols (Fig. 5F), often weakly differentiated and inconspicuous but sometimes with strongly elongated apices; hyphal pegs (Fig. 5G) common; heavily encrusted, thorny generocystidia (Fig. 6D) originating from subhymenial hyphae and emerging through hymenium, common especially in older parts of hymenium but sometimes forming amongst dissepiment edge hyphae; basidia (Fig. 6E–G) (5–)6–9(–10) ×(2.2–)2.7–3.7(–4) µm wide, tetrasterigmatic.

**Figure 7.**
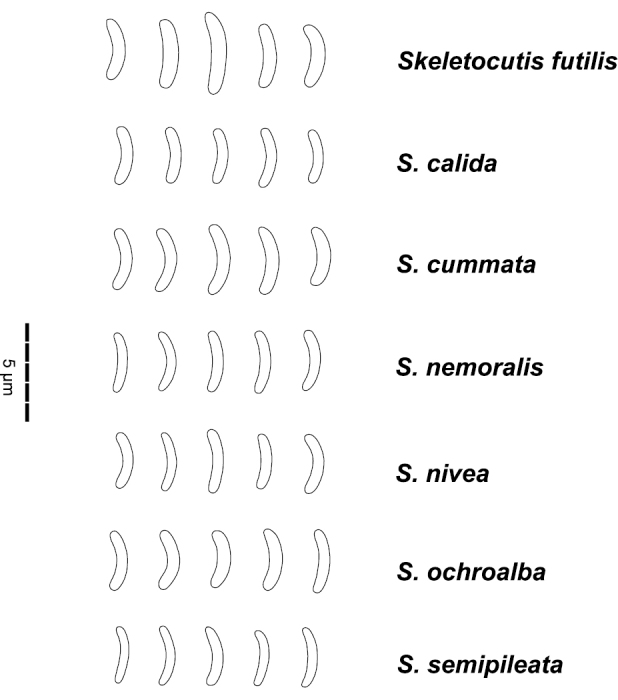
Spores of selected species in the *Skeletocutis nivea* complex.

**Basidiospores** (Fig. 7) narrowly allantoid, 2.5–4.0×0.4–0.9 µm, Q’=3.4–7.0, IKI-, CB- (contents CB+).

#### Discussion.

The tramal hyphal structure in *S. nivea* and *S. ochroalba* has traditionally been described as monomitic. However, our microscopic study revealed two distinct hyphal types existing in the trama of all species in the *S. nivea* complex. Amongst the normal clamped and thin-walled generative hyphae, there are usually at least some notably wider and slightly thick-walled hyphae which seem to lack clamps. We call these special hyphae tramal skeletal hyphae. They appear to originate from the generative hyphae in the trama and reach down almost to the pore mouths. Usually the lack of clamps, greater width and thicker walls help to tell them apart from generative hyphae in the trama. Although the tramal skeletal hyphae are usually wide and only slightly thick-walled, some specimens of *S. nivea* had narrower and solid skeletal hyphae in the trama.

Sometimes the tramal hyphal structure is dominated by the skeletal hyphae but sometimes they seem to be missing completely or occur only sporadically in otherwise monomitic tramal structure (at least in *S. nemoralis* and *S. semipileata*). They can also be difficult to detect when the whole tramal structure becomes sclerified and generative hyphae also develop thickened walls, which was observed in some specimens of *S. nivea*. In general, clear detection of tramal skeletal hyphae is easiest in a squash mount from very thin longitudinal slices of the tube layer which have been properly thinned to an almost disintegrated state.

The nature of the arbuscule-like ‘binding hyphae’ has been discussed by David (1982) and Niemelä (1985) and both express some reservations about using the term ‘trimitic’ to describe the *S. nivea* complex. They point out that the ‘binding hyphae’ in the morphospecies *S. nivea* and *S. ochroalba* originate as clampless side-branches of the generative hyphae and, hence, they are not binding hyphae proper, such as those of *Trametes*. David (1982) studied the staining reactions of the hyphal walls and noted that the walls of the ‘binding hyphae’ are congophilic and non-metachromatic whereas the walls of the skeletal hyphae are non-congophilic and metachromatic. Our observations confirm that all species in the *S. nivea* complex appear to be similar in this respect.

### Skeletocutis calida

Taxon classificationFungiPolyporalesPolyporaceae

Miettinen & A. Korhonen sp. nov.

822242

Figure 7

#### Holotype.

U.S.A. Florida: Alachua County, Gainesville, indet. angiosperm wood, 20 Nov 2013 Miettinen 17761 (H 7008665, isotype FLAS).

#### Description.

**Basidiocarps** annual; half-resupinate; up to 1.5 cm wide and 2 mm thick; hard when dry but easy to break apart; pilei thin, protruding up to 5 mm; margin incurved; upper surface minutely rough, matted, white to cream coloured when young, turning ochraceous; context up to 1.5 mm thick, faintly zonate in longitudinal section with thin dark lines separating layers of growth; tube layer up to 0.5 mm thick; pores 8–10(–11) per mm.

**Hyphal structure**: skeletal hyphae in context / subiculum (1.0–)2.0–2.9(–3.5) µm wide, in trama (1.0–)2.0–4.1(–5.2) µm wide, generative hyphae in trama 1.0–2.0(–2.9) µm wide.

**Basidiospores** 2.5–3.1(–3.3)×0.5–0.6(–0.7) µm, L=2.86 µm, W=0.55 µm, Q’=(4.0–)4.3–6.0(–6.9), Q=5.18, n=60/2.

#### Distribution and ecology.

The species is known only from two specimens from southern U.S.A., collected from warm temperate deciduous forests where specimens were growing on rather thin twigs of unidentified woody angiosperm.

#### Etymology.

Calidus (Lat.), warm, refers to the southern distribution.

#### Specimens examined.

U.S.A. Arkansas: Marion County, Yellville, indet. angiosperm wood, 25 Oct 2013 Miettinen 17466 (H, FLAS); Florida: (holotype, see above).

### Skeletocutis coprosmae

Taxon classificationFungiPolyporalesPolyporaceae

(G. Cunn.) A. Korhonen & Miettinen comb. nov.

822243

#### Basionym.

*Poria coprosmae* G. Cunn., Bulletin of the New Zealand Department of Industrial Research 72: 38 (1947).

#### Holotype.

New Zealand. Westland: Lake Mapourika, *Coprosma*, Nov 1946 J.M.Dingley (PDD 5252, studied).

#### Description.

**Basidiocarps** possibly perennial; resupinate to half-resupinate; up to 6 cm wide and 8 mm thick; hard when dry, breaking apart neatly; pilei fleshy, protruding up to 1.7 cm; margin blunt with narrow, sterile ridge on the underside; upper surface minutely rough, matted, white to cream coloured when young, turning ochraceous brown and finally blackish with age; pore surface sometimes with greenish-grey tints deep within the tubes in pileate part; context and subiculum whitish-cream colour to light greyish-brown near contact with substrate (in thick basidiocarps); context up to 5 mm thick, azonate; tube layer from 0.5–1.5 up to 6 mm thick and zonate in perennial basidiocarp, lighter horizontal zones appear where tubes are filled with arbuscule-like ‘binding hyphae’; pores (6–)7–8(–9) per mm.

**Hyphal structure**: skeletal hyphae in context 2.0–4.3(-5.3) µm wide, in subiculum (1.0–)2.0–3.5(–4.2) µm wide, in trama 2.0–4.0(–5.0) µm wide, generative hyphae in trama 1.0–2.3(–3.0) µm wide.

**Basidiospores** 2.8–3.2(–3.3)×0.5–0.7 µm, L=2.98 µm, W=0.57 µm, Q’=(4.0–)4.3–6.0(–6.4), Q=5.19, n=60/2.

#### Distribution and ecology.

Available material is very limited but suggests a rather wide, temperate Australasian distribution from Tasmania to southern New Zealand.

#### Specimens examined.

AUSTRALIA. Tasmania: Huon Valley, indet. angiosperm wood, 21 Nov 2006 Gates 1898 (H). NEW ZEALAND. Westland: (holotype, see above).

#### Discussion.

After examining the type, we have chosen to use a previously published name *Poria coprosmae* as the basionym for this Australasian species. *P. coprosmae* was described by Cunningham (1947) from Westland, New Zealand. He (Cunningham 1965) later concluded that his *P. coprosmae* was the same as *Polyporus semipileatus* Peck but he treated them mistakenly under the name *Tyromyces chioneus* (Fr.) P. Karst., as explained by Buchanan and Ryvarden (1988).

In their type studies of *Polyporaceae* species described by Cunningham, Buchanan and Ryvarden (1988) place *P. coprosmae* in the genus *Ceriporiopsis* Dom., rejecting placement in *Incrustoporia* or *Skeletocutis* based on the absence of encrusted hyphae. Rajchenberg (1995), on the other hand, found the hyphal structure in the holotype and other collections of *P. coprosmae* in PDD more in line with that of *S. nivea*. Our studies of the holotype confirm this view with the addition that we also observed encrusted generocystidia and thin-walled skeletal hyphae in the trama, which are characteristic for the *S. nivea* complex. Macroscopically, the only other studied specimen from Tasmania looks quite different from the type. However, we could not identify any clear microscopic differences and cannot rule out the possibility that the macroscopic differences represent variation between developmental stages. Nevertheless, considering the level of crypticity in the *S. nivea* complex, we have reservations in stating that our sequenced specimens are truly conspecific with the old type. Thus, we refrain from assigning an epitype for now.

*S. nivea* occurs in the North Island of New Zealand and it is possible that these two species could overlap as *S. nivea* has been shown to extend respectively far into the temperate zone in the northern hemisphere. The type specimen is a thin and resupinate basidiocarp on a fallen branch of a *Coprosma* shrub. The Tasmanian specimen, on the other hand, has evidently been growing on coarse wood and is unique in having a clearly perennial habit with a zonate tube layer.

### Skeletocutis cummata

Taxon classificationFungiPolyporalesPolyporaceae

A. Korhonen & Miettinen sp. nov.

822244

Figures 6G , 7

#### Holotype.

China. Jilin: Antu, Changbai Mountains, alt. 1300 m, *Picea jezoënsis*, 18 Sep 1998 Niemelä 6408 & Dai (H 7008666).

#### Description.

**Basidiocarps** annual; half-resupinate to pileate; up to 3 cm wide and (pilei) up to 1 cm thick; hard when dry but easy to break apart; pilei nodulous or thick but steeply sloping, protruding up to 1 cm; margin of pileus curved downwards, blunt, with narrow, woolly ridge on the underside; upper surface matted to minutely pubescent, white to cream coloured when young, turning ochraceous with almost orange hues; pore surface with ochraceous or sometimes salmon/peach coloured tints, sometimes a greenish-grey tint is visible in the tubes; context and subiculum finally coriaceous but looser and fibrous near cap edge and surface; context faintly zonate in longitudinal section with thin dark lines separating layers of growth; tube layer up to 1 mm thick; pores (5–)7–8(–13) per mm.

**Hyphal structure**: the outer layer of context typically with a loose, fibrous texture composed of radially orientated encrusted hyphae (tomentum). Skeletal hyphae in context / subiculum (1.0–)2.0–3.5(–5.0) µm wide, in trama 2.0–4.0(–5.0) µm wide, generative hyphae in trama 1.0–2.0(–2.6) µm wide.

**Basidiospores** (2.8–)2.9–3.4(–3.9)×0.5–0.8(–0.9) µm, L=3.1 µm, W=0.66 µm, Q’=(3.3–)3.8–6.0(–6.6), Q=4.68, n=270/9.

#### Distribution and ecology.

Boreal, Eurasian taiga; known from Fennoscandia, Czech Republic and Far East. The species seems to be rather rare in Europe but possibly more common in the Far East where Spirin (H) has collected it abundantly. The species has been found growing on fallen spruce logs (*Picea abies*, *P. jezoënsis*) but also on *Abies nephrolepis* and *Larix* sp.

#### Etymology.

Cummatus (Lat.), resinous, refers to the brown upper surface of basidiomes.

#### Specimens examined.

CHINA. Jilin: (holotype, see above). FINLAND. Etelä-Häme: Hämeenlinna, *P. abies* (fallen, fairly thin, still corticated tree), 17 Sep 2013 Niemelä 9088 & Spirin (H). RUSSIA. Khabarovsk Reg.: Khabarovsk Dist., Levyi Ulun, *P. jezoënsis*, 21 Aug 2012 Spirin 5472 (H); 5484 (H); Malyi Kukachan, *Larix* sp., 19 Aug 2012 Spirin 5430 (H); Malyi Niran, *P. jezoënsis*, 6 Aug 2012 Spirin 4897 (H); Ulun, *P. jezoënsis*, 26 Aug 2012 Spirin 5676 (H); Solnechny Dist., Igdomi, *Abies nephrolepis*, VIII.2011 Spirin 3857; Suluk-Makit, *P. jezoënsis*, 17 Aug 2011 Spirin 4170 (H).

#### Discussion.

*S. cummata* is most notably distinguished from other Eurasian species in the *S. nivea* complex by its occurrence on conifer wood. Spores of *S. cummata* are also larger than those of angiosperm-dwelling species apart from *S. futilis*. The pubescence on pileus surface in pileate specimens provides an additional identification cue. Very wide but thin-walled tramal skeletal hyphae seem to be particularly pronounced in this species. All distinctive features of *S. cummata* are shared with the North American conifer-dwelling species *S. ochroalba*.

### Skeletocutis futilis

Taxon classificationFungiPolyporalesPolyporaceae

Miettinen & A. Korhonen sp. nov.

822245

Figure 7

#### Holotype.

Finland. Uusimaa: Helsinki, *Sorbus aucuparia*, 24 Sep 2012 Miettinen 15745 (H 7008667).

#### Description.

**Basidiocarps** annual; half-resupinate; small, up to 5 mm wide and 1.5 mm thick; hard when dry but easy to break apart; pilei very small and nodulous; upper surface white when young, turning yellowish-brown; context and subiculum white; tube layer up to 0.2 mm thick; pores 6–8 per mm.

**Hyphal structure**: skeletal hyphae in context / subiculum (1.0–)2.0–3.0(–3.3) µm wide, in trama scarce, (1.0–)2.0–3.9(–4.9) µm wide, generative hyphae in trama 1.0–2.0(–3.2) µm wide.

**Basidiospores** 3.0–4.0×0.7–0.9 µm, L=3.33 µm, W=0.81 µm, Q’=(3.3–)3.4–5.1, Q=4.13, n=30.

#### Distribution and ecology.

The species is known only from the type specimen which was collected from a *Betula* stand on a disturbed site near the seashore in Helsinki, Finland (hemiboreal zone) where it was growing on rather thin twigs of *Sorbus aucuparia*.

#### Etymology.

Futilis (Lat.), fragile, insignificant.

#### Specimen examined.

FINLAND: Uusimaa: (holotype, see above).

#### Discussion.

While macroscopic features may be quite scanty, characteristic trimitic-looking subiculum, skeletal hyphae in trama and encrustations of dissepiment edge hyphae reveal *S. futilis* to be a member of the *S. nivea* complex. *S. futilis* can be distinguished from other species in the complex by thicker spores.

In our analyses, *S. futilis* constitutes a sister taxon to the rest of the *S. nivea* complex. The clade also includes S. aff. futilis in North America. Owing to the limited material available, we refrain from judging whether they represent geographic variation within one species or vicariant sister species. The voucher specimens of S. aff. futilis (Lindner DLL2009-067; -068 (CFMR)) are in a rather poor condition, but it seems that the small size of basidiocarps and thick spores are as characteristic for S. aff. futilis as they are for *S. futilis*.

### Skeletocutis impervia

Taxon classificationFungiPolyporalesPolyporaceae

Miettinen & A. Korhonen sp. nov.

822246

#### Holotype.

Uganda. Western Reg.: Kabale Dist., Bwindi Impenetrable National Park, indet. angiosperm wood, 18 Nov 2002 Ipulet F1104 (O 918073, isotype H 7017125).

#### Description.

**Basidiocarps** annual; half-resupinate; up to 6 mm thick; hard when dry, breaking apart neatly; pilei fleshy, protruding up to 5 mm; margin blunt; upper surface almost smooth, matted, white to cream coloured when young, turning ochraceous brown and finally blackish with age; context and subiculum whitish-cream to light greyish-brown; context up to 5 mm thick, zonate in longitudinal section with thin dark lines separating layers of growth; tube layer from up to 1 mm thick; pores (7–)8–9(–10) per mm.

**Hyphal structure**: skeletal hyphae in context 2.0–3.2(–4.1) µm wide, in subiculum (1.0–)2.0–3.0(–3.8) µm wide, in trama (1.0–)2.0–3.5(–4.9) µm wide, generative hyphae in trama 1.0–2.0 µm wide.

**Basidiospores** (2.8–)2.9–3.1×0.5–0.8 µm, L=2.97 µm, W=0.61 µm, Q’=(3.6–)3.8–6.0(–6.2), Q=4.85, n=30.

#### Distribution and ecology.

The species is known only from the type specimen, collected from Bwindi Impenetrable National Park in Uganda where it was reportedly growing on rotting branches.

#### Etymology.

Impervius (Lat.), impenetrable; the species is morphologically indistinguishable from its kins.

#### Specimen examined.

UGANDA. Western Reg.: (holotype, see above).

### Skeletocutis ipuletii

Taxon classificationFungiPolyporalesPolyporaceae

Miettinen & A. Korhonen sp. nov.

822247

#### Holotype.

Uganda. Western Reg.: Kabarole Dist., Kibale National Park, indet. angiosperm wood (leaning dead branch), 28 Oct 2002 Ipulet F761 (H 7017127, isotype O 918074).

#### Description.

**Basidiocarps** annual; half-resupinate; up to 2.5 cm wide and 6 mm thick; hard when dry, breaking apart neatly; pilei fleshy, protruding up to 5 mm; margin blunt; upper surface almost smooth, matted, white to cream coloured when young, turning ochraceous brown; pore surface cream coloured with greyish tint deep within the tubes; context and subiculum whitish-cream to light greyish-brown; context up to 5 mm thick, faintly zonate in longitudinal section with thin dark lines separating layers of growth; tube layer up to 1.5 mm thick; pores 9–10(–11) per mm.

**Hyphal structure**: skeletal hyphae in context / subiculum 2.0–3.0(–3.6) µm wide, in trama (1.0–)2.0–4.1(–5.0) µm wide, generative hyphae in trama 1.0–2.0(–2.2) µm wide.

**Basidiospores** 2.8–3.4×0.5–0.7(–0.8) µm, L=2.96 µm, W=0.6 µm, Q’=(4.0–)4.1–6.0(–6.2), Q=4.97, n=30.

#### Distribution and ecology.

The species is known only from the type specimen, collected from Kibale National Park in Uganda, where it was reportedly growing on a leaning, dead branch.

#### Etymology.

Named in honour of the pioneering Ugandan mycologist Perpetua Ipulet, who collected the type of this species.

#### Specimen examined.

UGANDA. Western Reg.: (holotype, see above).

### Skeletocutis lepida

Taxon classificationFungiPolyporalesPolyporaceae

A. Korhonen & Miettinen sp. nov.

822248

Figure 6A

#### Holotype.

Japan. Kansai Reg.: Shiga Prefecture, Otsu, indet. angiosperm wood, 7 Nov 2013 Schigel 7684 & Nakamori, Tanaka, Nakano (H 7008661, isotype TFM).

#### Description.

**Basidiocarps** annual; half-resupinate; pilei up to 3 cm wide, nodulous or up to 4 mm thick and fleshy, blunt edged; upper surface slightly rough, matted, white to cream coloured when young, turning ochraceous; context faintly zonate in longitudinal section with thin dark lines separating layers of growth; tube layer up to 1 mm thick; pores (7–)8–9(–10) per mm.

**Hyphal structure**: skeletal hyphae in context / subiculum (1.0–)2.0–3.3(–4.0) µm wide, in trama (1.0–)2.0–3.5(–4.9) µm wide, generative hyphae in trama 1.0–2.2(–3.0) µm wide.

**Basidiospores** See general description of microscopic structures; (2.8–)2.9–3.0(–3.1)×0.5–0.6 µm, L=2.95 µm, W=0.55 µm, Q’=4.8–6.0, Q=5.36, n=90/3.

#### Distribution and ecology.

Available material is limited but suggests a temperate East Asian distribution. Russian specimens represent rather small basidiocarps that were collected from thin, fallen angiosperm branches. The holotype from Japan is a sturdier basidiocarp that was probably growing on a thick branch or a log.

#### Etymology.

Lepidus (Lat.), charming, nice, elegant.

#### Specimens examined.

JAPAN. Kansai Reg.: (holotype, see above). RUSSIA. Khabarovsk Reg.: Khabarovsk Dist., Malyi Niran, *Tilia amurensis*, 7 Aug 2012 Spirin 4989 (H); Solnechny Dist., Boktor, *Quercus mongolica*, 8 Aug 2011 Spirin 3964 (H).

### Skeletocutis nemoralis

Taxon classificationFungiPolyporalesPolyporaceae

A. Korhonen & Miettinen sp. nov.

822249

Figures 4A–B , 6C , 7

#### Holotype.

Finland. Åland: Lemland, Nåtö, *Fraxinus excelsior* (fallen branch), 9 Oct 2012 Korhonen 90 (H 7008662).

**Basidiocarps** annual; resupinate to half-resupinate; bone hard when dry, breaking apart neatly; resupinate basidiocarps up to 10+ cm wide; pilei nodulous to shelf-shaped, often laterally fused and fleshy, protruding up to 1.3 cm; sterile margin often quite pronounced especially in resupinate part; upper surface slightly rough, matted, white to cream coloured when young, turning ochraceous and finally blackish with age; pore surface sometimes with a greenish-grey or turquoise tint emerging within the tubes especially in the pileate part but sometimes in scattered blotches; context sometimes faintly zonate in longitudinal section; tube layer up to 2 mm thick; pores (6–)7–8(–10) per mm.

**Hyphal structure**: skeletal hyphae in context / subiculum (1.0–)2.0–3.0(–4.0) µm wide, in trama (1.0–)2.0–3.9(–5.0) µm wide, generative hyphae in trama 1.0–2.2(–2.9) µm wide.

**Basidiospores** (2.8–)2.9–3.2(–4.0)×(0.4–)0.5–0.6(–0.7) µm, L=3.04 µm, W=0.56 µm, Q’=(4.1–)4.8–6.3(–7.8), Q=5.47, n=390/13.

#### Distribution and ecology.

Temperate Eurasia, found in Europe, Iran and Japan; on angiosperm wood, especially *Fraxinus*, preferring coarse substrates like thick branches or even logs.

#### Etymology.

Refers to the distribution area of the species in the nemoral zone.

#### Specimens examined.

FINLAND. Åland: Jomala, Ramsholm, *Fraxinus excelsior* (fallen branch), 10 Oct 2012 Korhonen 100 (H); *103* (H); Lemland, Nåtö, *F. excelsior* (fallen branch) 9 Oct 2012 Korhonen 86 (H); 89 (H); (holotype, see above); *Corylus avellana* (fallen branch) Korhonen 93 (H). NORWAY. Møre og Romsdal: Sunndal, *Populus tremula*, 24 Sep 2008 Gaarder 5257 (O 288578); Nord-Trøndelag: Verdal, *Sorbus aucuparia*, 30 Aug 2006 Klepsland JK06-S080 (O 284195); Sogn og Fjordane: Aurland, *Tilia cordata*, 10 Jul 2004 Brandrud 149-04 (O 166204). POLAND. Podlaskie Voivodeship: Białowieża National Park, *Carpinus betulus* (fallen branch), 15 Sep 2012 Korhonen 35 (H); *F. excelsior* (fallen branch), 15 Sep 2012 Korhonen 28 (H); 31 (H); 18 Sep 2012 Korhonen 83 (H).

#### Discussion.

*S. nemoralis* shares its wide distribution in Eurasia with similar-looking *S. semipileata.* Both species tend to form rather large, half-resupinate basidiocarps with fleshy pilei. *S. nemoralis* has slightly larger spores and pores than *S. semipileata*, but the distinction is probably too small for definitive identification.

In Europe, *S. nemoralis* does not reach as far to the northeast as *S. semipileata* and appears to be missing in continental Finland. At its north-eastern outpost in Åland Islands, *S. nemoralis* is rather common, especially in old coppice meadows where it prefers the wood of *Fraxinus*.

### Skeletocutis nivea

Taxon classificationFungiPolyporalesPolyporaceae

(Jungh.) Jean Keller, Persoonia 10(3): 353 (1979).

Figures 4C–D , 6D–E , 7

#### Basionym.

*Polyporus niveus* Jungh. Praemissa in floram cryptogamicam Javae insulae: 48 (1838).

#### Holotype.

Indonesia. Central Java: Mount Merapi, Junghuhn 44 (L).

#### Epitype.

Indonesia. Central Java: Mount Lawu, alt. 2130 m, old-growth montane forest dominated by *Castanopsis javanica*, indet. angiosperm wood (fallen branch), 22 May 2014 Miettinen 18217 (BO, designated here, duplicate H 7008663). MycoBank No. MBT378098

#### Description.

**Basidiocarps** annual; half-resupinate; hard when dry, breaking apart neatly; pilei nodulous to shelf-shaped, sometimes laterally fused and quite fleshy, up to 2 cm wide and 5 mm thick, protruding up to 1.3 cm, often connected to wider resupinate part; upper surface almost smooth to slightly rough, matted, white to cream coloured when young, turning ochraceous and finally blackish with age; pore surface often with a greenish-grey or turquoise tint emerging within the tubes particularly in the pileate part but often in scattered blotches; context and subiculum coriaceous, white; context sometimes faintly zonate in longitudinal section; tube layer up to 1 mm thick; pores (7–)8–10(–13) per mm.

**Hyphal structure**: trama dimitic but sometimes seemingly monomitic with slightly sclerified generative hyphae or sometimes clearly dimitic with solid skeletal hyphae; skeletal hyphae in context / subiculum (1.0–)2.0–3.0(–3.9) µm wide, in trama (1.0–)2.0–3.5(–4.9) µm wide, but only 2–3 µm wide and solid in specimens from New Zealand, generative hyphae in trama 1.0–2.3(–2.8) µm wide.

**Basidiospores** (2.7–)2.8–3.2(–3.7)×0.5–0.7(–0.8) µm, L=2.96 µm, W=0.56 µm, Q’=(3.9–)4.3–6.0(–6.2), Q=5.27, n=125/5.

#### Distribution and ecology.

From tropical southeast Asia to subtropical New Zealand in the south and temperate China in the north, on angiosperm wood.

#### Specimens examined.

CHINA. Jilin: Antu, Changbai Mountains, alt. 1100 m, *Alnus* sp. (fallen tree crown), 27 Aug 2015 Miettinen 10579.1 (H). INDONESIA. Central Java: (epitype, see above); alt. 2180 m, old-growth montane forest dominated by *Castanopsis javanica*, indet. angiosperm wood (fallen tree), 22 May 2014 Miettinen 18255 (ANDA, H); (holotype, see above). MALAYSIA. Sabah: Kinabalu Park, alt. 1675 m, lower montane forest, indet. angiosperm wood, 17 Jun 2013 Miettinen 16350 (SNP, H). NEW ZEALAND. Auckland: Hunua Ranges, indet. angiosperm wood, 19 Mar 1996 Ryvarden 38171 (O 916495); 38177 (O 916496).

#### Discussion.

The holotype of *S. nivea* is sterile but it possesses the encrusted generocystidia and arbuscule-like ‘binding hyphae’ characteristic to the *S. nivea* complex. Specimens from New Zealand represent a disjunct population and exhibit aberrant hyphal morphology with clearly dimitic trama. However, they do not stand out phylogenetically (in ITS) from the rest of *S. nivea*.

### Skeletocutis ochroalba

Taxon classificationFungiPolyporalesPolyporaceae

Niemelä, Naturaliste Canadien 112: 466 (1985).

Figures 5 , 7

#### Holotype.

Canada. Northern Quebec: Poste-de-la-Baleine, *Picea* sp., 7 Aug 1982 Niemelä 2695 (H 7017091)

#### Description.

**Basidiocarps** annual or possibly perennial; half-resupinate to pileate; up to 3 cm wide and (pilei) up to 8 mm thick; hard when dry but easy to break apart; pilei nodulous or thick but steeply sloping, protruding up to 1 cm; margin of pileus curved downwards, blunt, with narrow, woolly, ridge on the underside; upper surface matted to minutely pubescent, white or cream coloured when young, turning ochraceous brown; pore surface cream coloured with ochraceous or sometimes salmon/peach coloured tints, sometimes a greenish-grey tint is visible in the tubes; context and subiculum finally coriaceous but looser and fibrous near cap edge and surface; context faintly zonate in longitudinal section with thin dark lines separating layers of growth; tube layer up to 1 mm thick, sometimes divided by a thin white layer where tubes are filled with arbuscule-like ‘binding hyphae’, otherwise pale buff; pores (6–)7–8(–10) per mm.

**Hyphal structure**: the outer layer of context typically has a loose, fibrous texture composed of radially orientated hyphae. Skeletal hyphae in context / subiculum (1.0–)2.0–3.5(–4.0) µm wide, in trama 2.0–4.6(–6.2) µm wide, generative hyphae in trama 1.0–2.2(–2.9) µm wide.

**Basidiospores** (2.8–)2.9–3.7(–4.0)×0.5–0.8 µm, L=3.1 µm, W=0.67 µm, Q’=3.8–6.0(–7.0), Q=4.65, n=70/3.

#### Distribution and ecology.

Boreal North America, possibly quite rare, findings from Northern Quebec and Alberta in Canada; growing on fallen *Picea* logs.

#### Specimens examined.

CANADA. Alberta: William A. Switzer Provincial Park, *Picea* sp., 24 Jul 2015 Spirin 8854a (H); 8854b (H); Northern Quebec: Poste-de-la-Baleine, *Picea* sp., 7 Aug 1982 Niemelä 2689.

#### Discussion.

*S. ochroalba* is most notably distinguished from other North American species of the *S. nivea* complex by its occurrence on conifer wood. Surface of the pileus is characteristically pubescent in this species. The spores of *S. ochroalba* are also thicker than those of North American angiosperm-dwelling species apart from S. aff. futilis. Resemblance to the Eurasian conifer-dwelling *S. cummata* is strong both in phenology and microscopic structure.

### Skeletocutis semipileata

Taxon classificationFungiPolyporalesPolyporaceae

(Peck) Miettinen & A. Korhonen comb. nov.

822250

Figures 4E ; 6B, F

#### Basionym.

*Polyporus semipileatus* Peck, Annual Report on the New York State Museum of Natural History 34: 43 (1881).

#### Lectotype.

U.S.A. New York: Catskill Mountains, *Acer spicatum*, Aug 1880 Peck (BPI 220657 ISOTYPE, designated here). MycoBank No. MBT381253

#### Epitype.

U.S.A. New York: Essex County, Huntington Wildlife Forest, Betula sp. (branch), 15 Aug 2012 Miettinen 15536 (H 7008664, designated here, duplicate in BPI). MycoBank No. MBT381348

#### Description.

**Basidiocarps** annual; resupinate to half-resupinate; hard when dry, breaking apart neatly; resupinate basidiocarps up to 10+ cm wide; pilei nodulous to shelf-shaped, often laterally fused, up to 4 mm thick and protruding up to 1.5 cm, sometimes rather fleshy but often thin and sharp-edged with slightly incurved margin or with narrow, sterile ridge on the underside; upper surface slightly rough, matted, white to cream coloured when young, turning ochraceous and finally blackish with age; pore surface cream coloured with ochraceous or rarely faint salmon coloured tints, often a greenish-grey or turquoise tint emerges within the tubes particularly in the pileate part, sometimes in scattered blotches; context and subiculum coriaceous, white; context sometimes faintly zonate in longitudinal section; tube layer up to 2 mm thick; pores (7–)8–9(–11) per mm.

**Hyphal structure**: skeletal hyphae in context / subiculum (1.0–)2.0–3.3(–4.3) µm wide, in trama (1.0–)2.0–3.9(–5.0) µm wide, generative hyphae in trama 1.0–2.1(–3.0) µm wide.

**Basidiospores** (2.3–)2.8–3.1(–3.3)×0.4–0.6(–0.7) µm, L=2.97 µm, W=0.55 µm, Q’=(4.1–)4.7–7.0(–7.5), Q=5.43, n=450/15.

#### Distribution and ecology.

Temperate holarctic, extending to south-boreal zone at least in Fennoscandia; on various angiosperm species, often on thin fallen branches but sometimes on coarse wood as well.

#### Specimens examined.

FINLAND. Uusimaa: Helsinki, indet. angiosperm (fallen branch), 9 Oct 2011 Miettinen 14917.4 (H); Kirkkonummi, *Prunus padus* (fallen tree), 24 Oct 2012 Miettinen 15835 (H). Etelä-Häme: Hämeenlinna, Lammi, *Corylus avellana* (fallen branch), 11 Sep 2002 Miettinen 6694 (H); Pohjois-Häme: Jyväskylä, Vuoritsalo, *Juniperus communis* (fallen trunk), 15 Jul 2017 Miettinen 21003 (H). NORWAY. Møre og Romsdal: Nesset, *Ulmus glabra*, 22 Sep 2006 Ryvarden 47279 (O 361851); Sogn og Fjordane: Luster, *C. avellana*, 25 Aug 2007 Gaarder 5136 & Dybwad (O 293503). POLAND. Podlaskie Voivodeship: Hajnówka, *C. avellana*, 18 Sep 2012 Korhonen 76 (H). RUSSIA. Khabarovsk Reg.: Khabarovsk Dist., Ilga, *Actinidia kolomikta*, 10 Aug 2012 Spirin 5142 (H); Nizhny Novgorod Reg.: Lukoyanov Dist., Sanki, *C. avellana*, 7 Aug 2005 Spirin 2326 (H). UNITED KINGDOM. Scotland: South Lanarkshire, *P. padus* (fallen tree), 6 Jul 2010 Miettinen 14114 (H). U.S.A. Massachusetts: Holden, *Betula* sp. (fallen branch), 6 Sep 2013 Miettinen 16823 (H); Minnesota: Waseca, *Tilia americana* (fallen branch), 18 Aug 2013 Miettinen 16693.1 (H); New York: (lectotype, see above); (epitype, see above); indet. angiosperm wood, 18 Aug 2012 Miettinen 15715 (H); *Betula* sp., 22 Sep 2013 Miettinen 17135 (H); *Fagus grandifolia* (fallen tree crown), 20 Sep 2013 Miettinen 17074 (H).

#### Discussion.

*S. semipileata* seems to be the most widespread species in the *S. nivea* complex and overlaps with many of the other angiosperm-dwelling species: in Europe with *S. futilis* and *S. nemoralis*; in the Far East with *S. lepida* and *S. nivea*; and in North America with S. aff. futilis and possibly with *S. calida*. Apart from *S.* (aff.) *futilis*, these species are almost impossible to distinguish from each other morphologically.

### Skeletocutis unguina

Taxon classificationFungiPolyporalesPolyporaceae

Miettinen & A. Korhonen sp. nov.

822251

Figure 4F

#### Holotype.

China. Yunnan: Xishuangbanna, Xishuangbanna Biosphere Reserve, alt. 700 m, indet. angiosperm wood, 5 Aug 2005 Miettinen 10002 (H 7008668, isotype BJFC).

#### Description.

**Basidiocarps** annual; half-resupinate; small, up to 1 cm wide and 1 mm thick; hard when dry but easy to break apart; pilei thin, protruding up to 4 mm; margin incurved; upper surface minutely rough, matted, white to cream coloured when young, turning ochraceous; context up to 0.7 mm thick, azonate; tube layer up to 0.3 mm thick; pores 7–9 per mm.

**Hyphal structure**: skeletal hyphae in context 1.0–2.9(–3.5) µm wide, in subiculum (1.0–)2.0–2.9(–3.2) µm wide, in trama 2.0–4.0(–4.8) µm wide, generative hyphae in trama 1.0–1.9(–2.0) µm wide.

**Basidiospores** 2.9–3.2(–3.3)×(0.4–)0.5–0.6(–0.7) µm, L=3.04 µm, W=0.55 µm, Q’=(4.6–)4.8–6.4(–7.5), Q=5.49, n=30.

#### Distribution and ecology.

The species is known only from the type specimen, collected from Yunnan, China, where it was growing as small individual pilei on thin twigs of unidentified woody angiosperm.

#### Etymology.

Derived from unguis (Lat.), claw, nail; refers to nail-like basidiome caps.

#### Specimen examined.

CHINA. Yunnan: (holotype, see above).

### Skeletocutis yuchengii

Taxon classificationFungiPolyporalesPolyporaceae

Miettinen & A. Korhonen sp. nov.

822252

#### Holotype.

China. Yunnan: Xishuangbanna, Menglun, alt. 640 m, indet. angiosperm wood, 4 Aug 2005 Miettinen 9950 (H 7008660, isotype BJFC, strain FBCC 1132).

#### Description.

**Basidiocarps** annual; resupinate to half-resupinate; small, up to 2.5 cm wide and 3 mm thick; hard when dry but easy to break apart; pilei nodulous, protruding up to 3 mm; margin blunt; upper surface minutely rough, matted, white to cream coloured when young, turning ochraceous; pore surface cream coloured with yellowish to ochraceous tints; context up to 2.7 mm thick faintly zonate in longitudinal section with fuzzy, ochraceous lines separating layers of growth; tube layer up to 0.3 mm thick; pores 8–10(–11) per mm.

**Hyphal structure**: skeletal hyphae in context / subiculum 1.0–2.9(–3.8) µm wide, in trama (2.0–)3.0–4.0(–5.1) µm wide, generative hyphae in trama 1.0–2.0(–2.2) µm wide.

**Basidiospores** (2.7–)2.8–3.1(–3.2)×(0.4–)0.5–0.7 µm, L=2.96 µm. W=0.59 µm, Q’=(4.0–)4.1–6.0(–7.2), Q=4.99, n=90/3.

#### Distribution and ecology.

The species is known from three specimens from Yunnan, China, collected from twigs of unidentified woody angiosperm.

#### Etymology.

In honour of the Chinese polypore researcher, Prof. Yu-Cheng Dai.

#### Specimens examined.

CHINA. Yunnan: (holotype, see above); Xishuangbanna Biosphere Reserve, alt. 700 m, indet. angiosperm wood (dead standing tree), 9 Aug 2005 Miettinen 10150.2 (H); Xishuang Banna Primeval Forest Park, indet. angiosperm wood (fallen branch), 16 Aug 2005 Miettinen 10366.1 (H).

##### Rejected names

### Poria hymeniicola

Taxon classificationFungiPolyporalesPolyporaceae

Murrill, Mycologia 12(6): 305 (1920).

#### Holotype.

U.S.A. Maine: Piscataquis Co., Medford, Camp Sunday, on dead *Populus*, 28 Aug 1905 Murrill (NY, studied).

#### Specimen examined.

U.S.A. Maine: (holotype, see above)

#### Discussion.

*P. hymeniicola* is a poorly known species from North America which has sometimes been associated with the *S. nivea* complex (*P. semipileatus* by Lowe (1947, 1966)). Niemelä (1998) studied the type specimen and concluded that the dimitic trama with solid skeletal hyphae does not match with the *S. nivea* complex. Even though we have observed some specimens of *S. nivea* with such hyphal structure, they were not observed in North American material. Furthermore, the basidiocarp of the type specimen grew on a dead basidiocarp of another polypore species, unlike any of our studied material of the *S. nivea* complex. The species would appear to be related to *S. stellae* and related species (*Incrustoporia*).

### Polyporus alboniger

Taxon classificationFungiPolyporalesPolyporaceae

Lloyd ex G. Cunn., Proceedings of the Linnean Society of New South Wales 75: 227 (1950).

#### Type.

Australia. Tasmania: Hobart, Rodway (BPI 301712).

#### Discussion.

Cunningham (1950) mentions the name *P. alboniger* as the label of a herbarium specimen he determined to be *P. atromaculus* (see below). We consider the name to be invalid as it lacks proper description (ICBN Melbourne Art. 38.1 & 39.1).

### Polyporus atromaculus

Taxon classificationFungiPolyporalesPolyporaceae

Lloyd, Bulletin of the Lloyd Library 35: 98 (1936).

#### Type.

Australia. Tasmania: Hobart, Rodway (BPI 302037).

#### Discussion.

Cunningham (1965) considered *P. atromaculus* to be a synonym of *S. nivea* (or *Tyromyces chioneus*, as he called it). The name refers to Tasmanian collections of L. Rodway. Lloyd (1922) used the name *P. atromaculatus* in reporting his determinations to Rodway. Referencing Lloyd (1922), Stevenson and Cash (1936) published *Polyporus atromaculus* Lloyd in herb. accompanied by a description and specification of a type specimen in a list of fungus names proposed by Lloyd. It is doubtful whether their intention was to validate the name but the description remains invalid (ICBN Melbourne Art. 39.1) and we reject the name. Ryvarden (1990) appears to share this view as he does not include *P. atromaculus* in his type studies of *Polyporus* species described by Lloyd.

## Supplementary Material

XML Treatment for Skeletocutis nivea

XML Treatment for Skeletocutis calida

XML Treatment for Skeletocutis coprosmae

XML Treatment for Skeletocutis cummata

XML Treatment for Skeletocutis futilis

XML Treatment for Skeletocutis impervia

XML Treatment for Skeletocutis ipuletii

XML Treatment for Skeletocutis lepida

XML Treatment for Skeletocutis nemoralis

XML Treatment for Skeletocutis nivea

XML Treatment for Skeletocutis ochroalba

XML Treatment for Skeletocutis semipileata

XML Treatment for Skeletocutis unguina

XML Treatment for Skeletocutis yuchengii

XML Treatment for Poria hymeniicola

XML Treatment for Polyporus alboniger

XML Treatment for Polyporus atromaculus

## Supplementary materials

Supplementary material 1 Specimens and INSDC accession numbers of DNA sequences used in this study Data type: molecular data Explanation note: Sequences retrieved from INSDC are denoted with*. This is an open access article distributed under the terms of the Creative Commons Attribution License (CC BY 4.0), which permits unrestricted use, distribution, and reproduction in any medium, provided the original author and source are credited.Aku Korhonen, Jaya Seelan Sathiya Seelan, Otto Miettinen

Supplementary material 2 Estimates of average genetic divergence over sequence pairs within species Data type: molecular data Explanation note: The number of base substitutions per site ×100 (%) obtained from averaging over all sequence pairs within each species are shown in first column, followed by standard error (SE) estimates (500 bootstrap replicates) and the number (N) of compared sequences are shown. This is an open access article distributed under the terms of the Creative Commons Attribution License (CC BY 4.0), which permits unrestricted use, distribution, and reproduction in any medium, provided the original author and source are credited.Aku Korhonen, Jaya Seelan Sathiya Seelan, Otto Miettinen

Supplementary material 3 Estimates of genetic divergence over ITS sequence pairs between species Data type: molecular data Explanation note: The number of base substitutions per site ×100 (%) obtained from averaging over all sequence pairs between species (1–14) are shown below the diagonal and standard error (SE) estimates (500 bootstrap replicates) above the diagonal. This is an open access article distributed under the terms of the Creative Commons Attribution License (CC BY 4.0), which permits unrestricted use, distribution, and reproduction in any medium, provided the original author and source are credited.Aku Korhonen, Jaya Seelan Sathiya Seelan, Otto Miettinen

Supplementary material 4 Estimates of evolutionary divergence over tef1 sequence pairs between species Data type: molecular data Explanation note: The number of base substitutions per site ×100 (%) obtained from averaging over all sequence pairs between species (1–13) are shown below the diagonal and standard error (SE) estimates (500 bootstrap replicates) above the diagonal. This is an open access article distributed under the terms of the Creative Commons Attribution License (CC BY 4.0), which permits unrestricted use, distribution, and reproduction in any medium, provided the original author and source are credited.Aku Korhonen, Jaya Seelan Sathiya Seelan, Otto Miettinen

Supplementary material 5 Distribution maps Data type: occurence Explanation note: Collection localities of specimens included in this study, indicating the approximate species distributions of the *Skeletocutis nivea* complex (A) in Europe and Western Asia, (B) in East Asia, Indomalaya and Australasia and (C) in North America. This is an open access article distributed under the terms of the Creative Commons Attribution License (CC BY 4.0), which permits unrestricted use, distribution, and reproduction in any medium, provided the original author and source are credited.Aku Korhonen, Jaya Seelan Sathiya Seelan, Otto Miettinen
